# Arnicolide C Suppresses Tumor Progression by Targeting 14-3-3θ in Breast Cancer

**DOI:** 10.3390/ph17020224

**Published:** 2024-02-08

**Authors:** Zhengrui Liu, Xiaodan Lyu, Jiaxu Chen, Benteng Zhang, Siman Xie, Yan Yuan, Li Sun, Shengtao Yuan, Hong Yu, Jian Ding, Mei Yang

**Affiliations:** 1 Jiangsu Key Laboratory of Drug Screening, China Pharmaceutical University, Nanjing 210009, China; 2 College of Pharmacy, Lanzhou University, Lanzhou 730000, China; 3 Department of Pharmacology, School of Pharmacy, China Pharmaceutical University, Nanjing 210009, China; 4 Jiangsu Center for Pharmacodynamics Research and Evaluation, China Pharmaceutical University, Nanjing 210009, China; 5 Department of Pathology, The Affiliated Taizhou People’s Hospital of Nanjing Medical University, Taizhou 225300, China; 6 State Key Laboratory of Drug Research, Shanghai Institute of Materia Medica, Chinese Academy of Sciences, Shanghai 201203, China

**Keywords:** arnicolide C, breast neoplasms, cell proliferation, 14-3-3θ, PDX

## Abstract

**Background:** Arnicolide C, which is isolated from *Centipeda minima*, has excellent antitumor effects. However, the potential impacts and related mechanisms of action of arnicolide C in breast cancer remain unknown. **Methods:** The viability of breast cancer cells was measured using MTT (3-(4,5-Dimethylthiazol-2-yl)-2,5-diphenyltetrazolium bromide) assay and colony formation assays. For analysis of apoptosis and the cell cycle, flow cytometry was used. A molecular docking approach was used to explore the possible targets of arnicolide C. Western blot analysis was used to detect changes in the expression of 14-3-3θ and proteins in related pathways after arnicolide C treatment in breast cancer cells. The anti-breast cancer effect of arnicolide C in vivo was evaluated by establishing cell-derived xenograft (CDX) and patient-derived xenograft (PDX) models. **Results:** Arnicolide C inhibited proliferation, increased apoptosis, and induced G1 arrest. In particular, molecular docking analysis indicated that arnicolide C binds to 14-3-3θ. Arnicolide C reduced 14-3-3θ expression and inhibited its downstream signaling pathways linked to cell proliferation. Similar results were obtained in the CDX and PDX models. **Conclusion:** Arnicolide C can have an anti-breast cancer effect both in vitro and in vivo and can induce cell cycle arrest and increase apoptosis in vitro. The molecular mechanism may be related to the effect of arnicolide C on the expression level of 14-3-3θ. However, the specific mechanism through which arnicolide C affects 14-3-3θ protein expression still needs to be determined.

## 1. Introduction

Breast cancer is a malignant tumor that occurs more frequently in women. As of 2020, the incidence of breast cancer has surpassed that of lung cancer, and breast cancer ranks first in global cancer incidence and global female cancer deaths. The newly diagnosed cases and deaths accounted for approximately 12.50% and 6.92%, respectively, of the overall incidence of and deaths from malignant tumors [1]. Currently, drug therapy for breast cancer mainly includes endocrine therapy, chemotherapy, and targeted therapy. Due to the high heterogeneity of breast cancer, breast cancer treatment strategies also vary across the subtypes. Surgical treatment combined with radiotherapy is an important clinical treatment approach for breast cancer. Patients with estrogen receptor (ER) and/or progesterone receptor (PR) positive invasive breast cancer should receive postoperative adjuvant endocrine therapy. Chemotherapy is mainly targeted towards patients with negative or low expression of ER/PR. Currently, there are many types of chemotherapeutic drugs, for example, anthracyclines, taxanes, vinorelbine, capecitabine, gemcitabine, and platinum. However, during chemotherapy, patients are prone to experiencing adverse reactions, and some complications may occur during treatment, threatening health and reducing the quality of life of patients. A limitation of the existing remedies is that these treatments may affect the quality of life through adverse effects and complications. Therefore, there is an urgent need to find new antitumor drugs. Traditional Chinese medicine (TCM) therapy, a critical adjuvant treatment approach for breast cancer, can reduce the adverse effects of radiotherapy, chemotherapy, and endocrine therapy while also mitigating cancer-related symptoms and improving quality of life.

*Centipeda minima* (*C. minima*) is a commonly used medicinal plant in China. Modern pharmacological research suggests that active compounds isolated from medicinal plants have multiple pharmacological effects [2,3,4]. At present, it has been reported that medicinal plants have been proven to have anti-inflammatory [5,6] and antibacterial [7,8] effects, according to traditional Chinese medicine books, and these compounds also have a wide range of antitumor effects, which can inhibit the proliferation of nasopharyngeal cancer [9], gastric cancer [10], colon cancer [11] and other tumors and induce tumor cell apoptosis. Arnicolide C is a sesquiterpene lactone [12] isolated from *C. minima*. Pharmacological studies have shown that arnicolide C has anti-inflammatory [13], neuroprotective [14], and antitumor effects [3,15]. A screen of the antitumor activity of arnicolide C revealed that this compound has a wide range of antitumor effects on nasopharyngeal cancer and cervical cancer and that it has a good inhibitory effect on liver cancer. However, its role in breast cancer and its related mechanisms have rarely been reported. Further exploration of the underlying mechanism of action of arnicolide C is needed to lay a foundation for its future development.

14-3-3 proteins exist as homodimers or heterodimers [16]. 14-3-3 proteins can bind to other proteins [17,18] and participate in various biological activities, including signal transduction, cell cycle progression, apoptosis, and enzyme activity regulation [19,20,21,22,23]. Studies have shown that 14-3-3 proteins are essential for cancer development [24,25,26,27,28,29]. 14-3-3 proteins are ubiquitously expressed in eukaryotic cells, and there are seven main protein isoforms in mammals [30]. Specifically, the isoforms are α/β, γ, ε, η, σ, δ/ζ, and θ/τ, with the α and δ isoforms being the phosphorylated forms of the β and ζ isoforms, respectively [30]. Among the isoforms, 14-3-3β is highly expressed in a variety of cancers. Studies have shown that 14-3-3β can promote the proliferation and migration of osteosarcoma and glioma cells [31,32]. 14-3-3γ is highly expressed in breast cancer and non-small cell lung cancer and is associated with shorter overall survival [24,33,34]. 14-3-3ε is highly expressed in various tumors, such as breast cancer, gastric cancer, and liver cancer, and is associated with poor prognosis [35,36,37]. 14-3-3η can promote tumor malignancy, and targeting 14-3-3η can inhibit tumor progression in cancers such as liver cancer [27,38,39]. 14-3-3σ is able to regulate cholangiocarcinoma cell survival through the phosphatidylinositol 3-kinase/serine-threonine kinase (PI3K/AKT) signaling pathway in hepatocellular carcinoma [40]. 14-3-3ζ is involved in the regulation of a large number of important pathways in tumors, and high expression of 14-3-3ζ is closely associated with poor survival in tumor patients [29,41,42]. 14-3-3θ can promote tumor survival in leukemia and glioma [43,44]. Compared with its expression in healthy tissues, 14-3-3θ is overexpressed in breast cancer tissues and plays a vital role in the metastasis and prognosis of breast cancer [45]. 14-3-3θ inhibition can affect the proliferation and metastasis of breast cancer cells via a mechanism related to inactivation of the Rapidly accelerated fibrosarcoma/extracellular regulated protein kinases (RAF/ERK), PI3K/AKT, and Janus kinase/signal transducer and activator of transcription (JAK/STAT) pathways [46,47,48]. Since 14-3-3θ plays an important role in the proliferation of cancer cells, new therapies targeting this protein have attracted increased amounts of attention.

We report a potential target of arnicolide C, a promising antineoplastic agent. We aimed to evaluate the antitumor effects of arnicolide C on breast cancer and explore the relevant molecular mechanisms involved. At present, the effects of arnicolide C on breast cancer have not been reported, and the mechanism of action of arnicolide C in tumors has rarely been studied. Hence, further research into both the impact of arnicolide C on inhibiting the proliferation of breast cancer cells and the underlying mechanism will contribute to the development of new drugs and more effective treatments for breast cancer.

## 2. Results

### 2.1. Arnicolide C Inhibited the Growth of Breast Cancer Cell Lines

**Figure 1:**
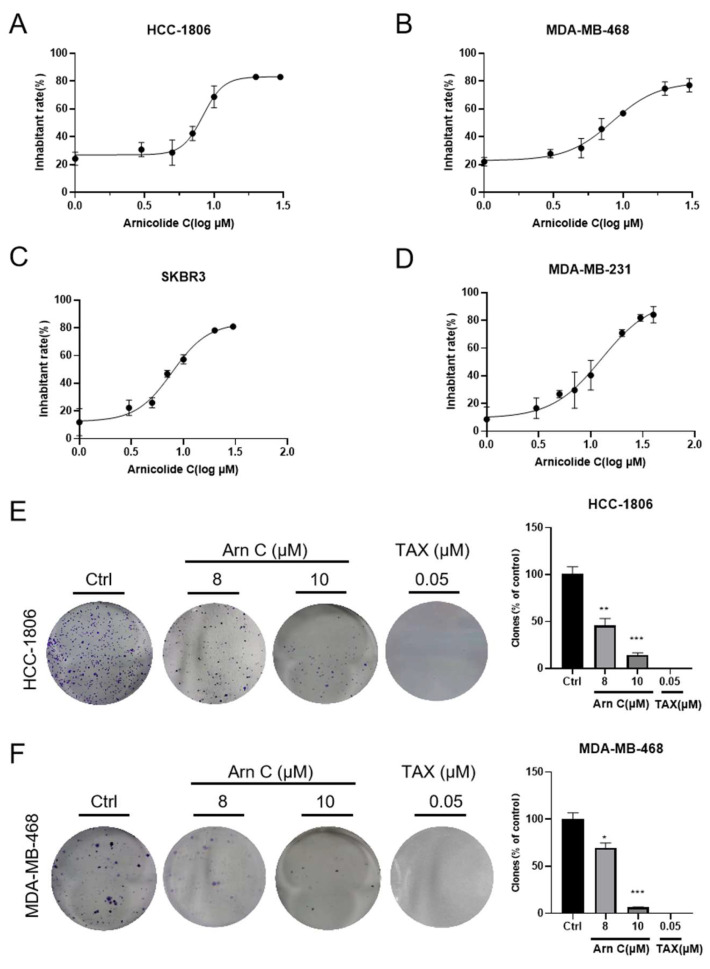
Effect of arnicolide C on breast cancer cell viability and colony formation. (**A**–**D**) HCC-1806, MDA-MB-468, MDA-MB-231, and SKBR3 cells were treated with arnicolide C (Arn C) for 72 h. An MTT assay was used to analyze cell viability. Cell viability was normalized to that of the control group for each cell line. (**E**,**F**) HCC-1806 and MDA-MB-468 cells were cultured for 48 h with arnicolide C (Arn C) (8 or 10 μM) or paclitaxel (TAX) (0.05 μM). The cells were cultured for seven days. The percentage of colonies was obtained for the HCC-1806 and MDA-MB-468 cells compared with that of the control group. Compared to the control group: * *p* < 0.05, ** *p* < 0.01, *** *p* < 0.001.

To explore the inhibitory effect of arnicolide C on breast cancer cells, we compared the viability of breast cancer cell lines (HCC-1806, MDA-MB-468, MDA-MB-231, and SKBR3) after treatment with arnicolide C. MTT assays indicated that arnicolide C inhibited cell growth (Figure 1A–D), with IC_50_ values of 8.50, 8.13, 14.51, and 8.02 μM. Then, HCC-1806 and MDA-MB-468 cells were selected for a colony formation assay. Treatment with 8 or 10 μM arnicolide C potently inhibited colony formation (Figure 1E,F). Thus, these results indicate that arnicolide C inhibited breast cancer progression in vitro.

### 2.2. Arnicolide C-Induced Apoptosis and Cell Cycle Arrest in Breast Cancer Cells

**Figure 2:**
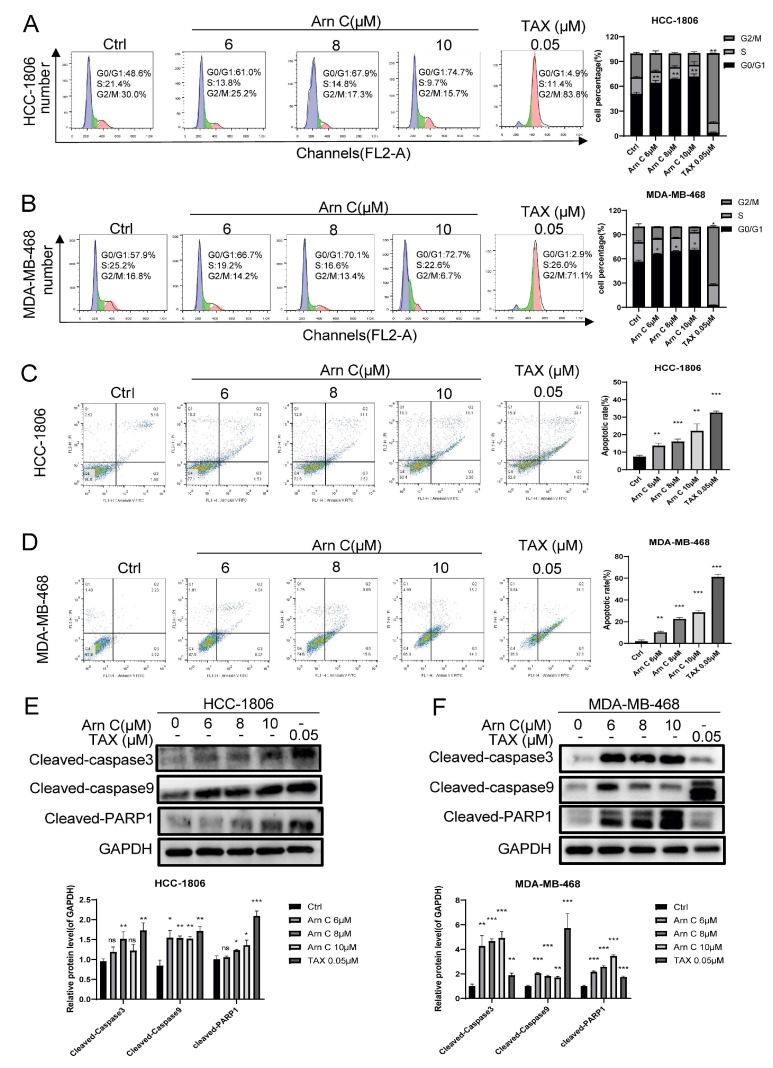
Arnicolide C induced apoptosis and G1 arrest in breast cancer cells. The cell cycle distribution of HCC-1806 (**A**) and MDA-MB-468 (**B**) cells was determined via PI staining. The graph shows the quantitative results of the percentage of cells in different cell cycle phases. HCC-1806 (**C**) and MDA-MB-468 (**D**) cells were cultured for 48 h with arnicolide C (Arn C) (0, 6, 8, or 10 μM) or paclitaxel (0.05 μM). Annexin V-FITC and PI staining was used to determine the cell cycle distribution of HCC-1806 and MDA-MB-468 cells. The graph shows the quantitative results of the apoptosis rate. (**E**,**F**) The protein levels of cleaved Caspase3, cleaved Caspase9, and cleaved PARP1 in HCC-1806 and MDA-MB-468 cells were measured. GAPDH was used as a loading control. Compared to the control group: * *p* < 0.05, ** *p* < 0.01, *** *p* < 0.001, ns (not significant).

We next evaluated the effect of arnicolide C on the cell cycle in breast cancer cells via flow cytometry (propidium iodide (PI) staining). Figure 2A,B show that arnicolide C induced G1 arrest in breast cancer cells in a dose-dependent manner. Annexin V/PI staining was performed after treatment with arnicolide C to detect apoptosis, and the results showed that the apoptosis rate increased in both HCC-1806 and MDA-MB-468 cells treated with arnicolide C compared to the control cells (Figure 2C,D). Cleaved Caspase3, cleaved Caspase9, and cleaved PARP1 have been identified as specific apoptosis markers. Apoptosis was further confirmed by the increased protein levels of cleaved Caspase3, Cleaved Caspase9, and cleaved PARP1 upon arnicolide C treatment (Figure 2E,F). The above results indicate that arnicolide C inhibits the proliferation of breast cancer cells by inducing apoptosis and G1 arrest.

### 2.3. Effects of Arnicolide C on 14-3-3θ

**Figure 3:**
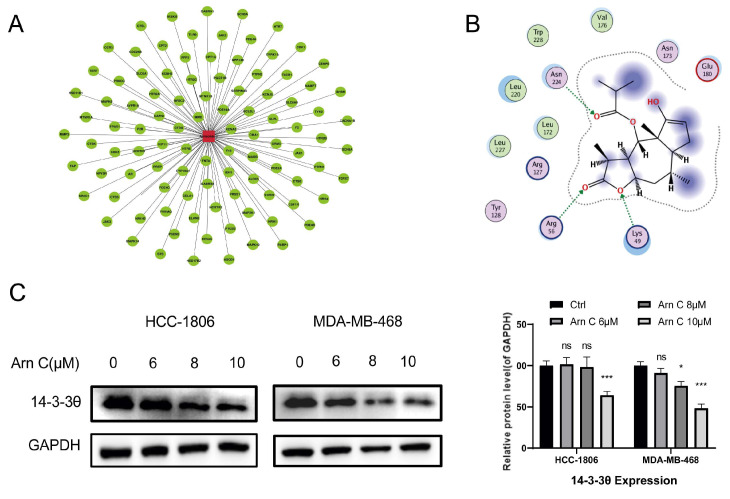
Effects of arnicolide C on 14-3-3θ. (**A**) Target prediction via http://www.swissdock.ch/docking# (accessed on 26 October 2023). (**B**) MOE software (2019.0102) was used to analyze the interaction between Arn C and the 14-3-3θ protein. (**C**) Cells were cultured for 48 h with arnicolide C (Arn C) (0, 6, 8, or 10 μM). The protein expression of 14-3-3θ was measured in HCC-1806 and MDA-MB-468 cells. GAPDH was used as a loading control. Compared to the control group: * *p* < 0.05, *** *p* < 0.001, ns (not significant). YWHAQ is the gene of the 14-3-3θ protein.

The potential targets of arnicolide C were analyzed using in silico molecular docking. By investigating the obtained potential target library, we found that 14-3-3θ is highly expressed in breast cancer and is associated with proliferation, metastasis, and poor prognosis. Then, 14-3-3θ was selected from the docking list as a potential target of arnicolide C (Figure 3A). Furthermore, to explore whether arnicolide C can bind to the 14-3-3θ protein, a protein–small molecule affinity analysis was performed using molecular docking. As shown in Figure 3B, the results suggested an interaction between arnicolide C and 14-3-3θ. This finding indicates that arnicolide C may affect the function of the 14-3-3θ protein in cells. Therefore, the 14-3-3θ protein level was measured in HCC-1806 and MDA-MB-468 cells after treatment with arnicolide C for 48 h. Our findings demonstrated that arnicolide C reduced the expression of 14-3-3θ, indicating that 14-3-3θ is a potential target of arnicolide C (Figure 3C).

### 2.4. Arnicolide C Inhibits Proliferation-Related Signaling Pathways in Breast Cancer Cells

**Figure 4:**
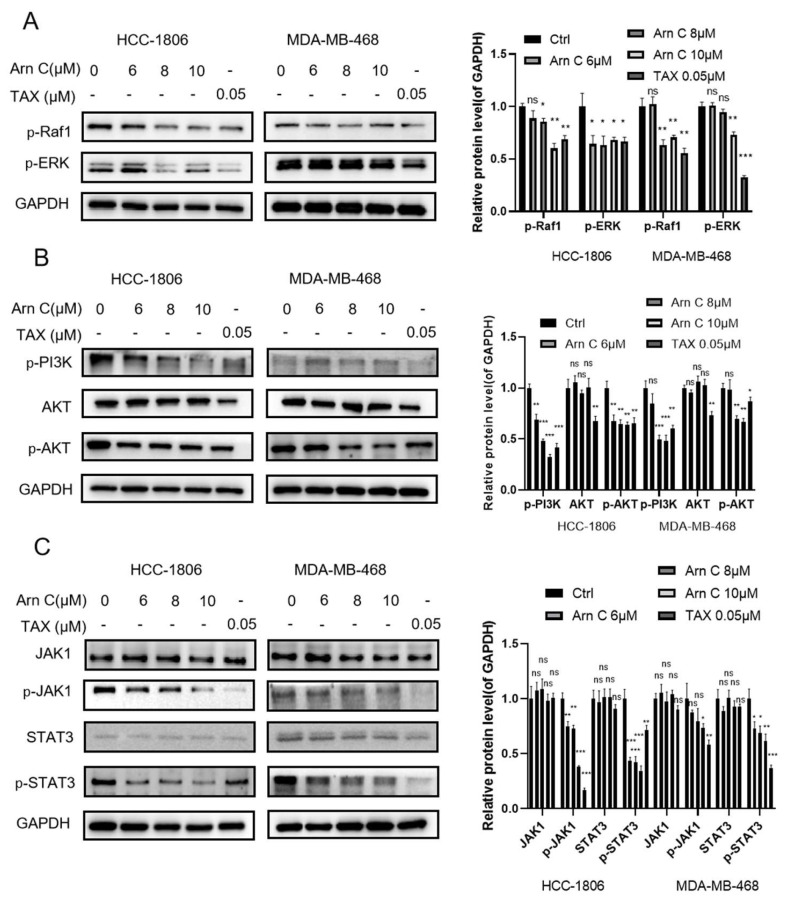
Expression of proteins in the RAF/ERK, PI3K/AKT, and JAK/STAT pathways after treatment with arnicolide C. (**A**–**C**) Cells were cultured for 48 h with arnicolide C (Arn C) (0, 6, 8, or 10 μM) or paclitaxel (0.05 μM). The protein levels of p-Raf1 (S338), p-ERK1/2, p-PI3K, p-AKT, p-JAK1, and p-STAT3 were measured in HCC-1806 and MDA-MB-468 cells. GAPDH was used as a loading control. Compared to the control group: * *p* < 0.05, ** *p* < 0.01, *** *p* < 0.001, ns (not significant).

The above results showed that arnicolide C can reduce the expression level of 14-3-3θ. However, the mechanism through which arnicolide C causes a decrease in the expression level of 14-3-3θ still needs further exploration. Next, the relevant mechanism of action of arnicolide C was further explored. 14-3-3θ is overexpressed in breast cancer and plays an essential role in the proliferation and metastasis of breast cancer cells. 14-3-3θ affects the proliferation and metastasis of breast cancer cells through the RAF/ERK, PI3K/AKT, and JAK/STAT pathways. Thus, we hypothesized that arnicolide C inhibits signaling pathways by targeting 14-3-3θ. As expected, Western blot data showed that treatment with arnicolide C (6 μM, 8 μM, or 10 μM) significantly reduced the levels of p-Raf1 (S338), p-ERK1/2, p-PI3K, p-AKT, p-JAK1, and p-STAT3 (Figure 4A–C). Thus, these results indicate that arnicolide C may inhibit proliferation-related signaling pathways by targeting 14-3-3θ.

### 2.5. Arnicolide C Exerted Antitumor Effects on Xenograft Tumors

**Figure 5:**
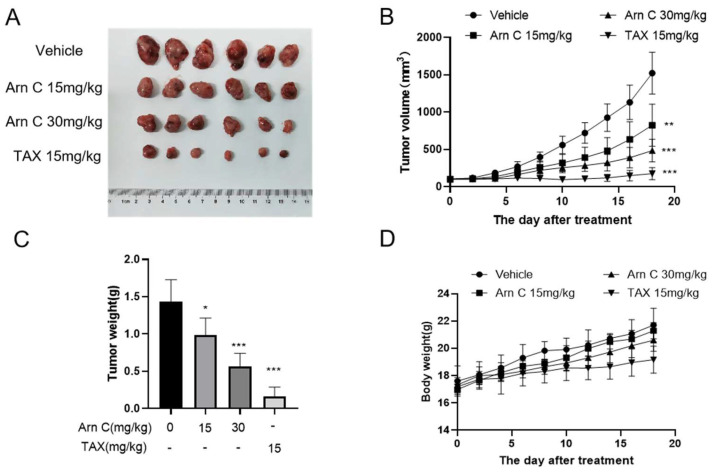
Arnicolide C inhibited the growth of MDA-MB-468 xenograft tumors in vivo. (**A**). Image of MDA-MB-468 xenograft tumors resected from mice. (**B**). The volume of the MDA-MB-468 xenograft tumors was measured after arnicolide C (Arn C) treatment. (**C**). The weights of the MDA-MB-468 xenograft tumors. (**D**). The body weights of the tumor model mice were measured after arnicolide C (Arn C) treatment. Compared to the control group: * *p* < 0.05, ** *p* < 0.01, *** *p* < 0.001).

The above results showed that arnicolide C suppressed breast cancer cell proliferation in vitro; hence, we investigated whether arnicolide C exerts its anti-breast cancer effects through 14-3-3θ in vivo. The xenograft assay with MDA-MB-468 cells in nude mice showed significant inhibition of tumor growth after treatment with arnicolide C (15 or 30 mg/kg, i.p.) (Figure 5A–C). Arnicolide C had no significant effect on the weight of the mice (Figure 5D).

### 2.6. Arnicolide C Exerted Antitumor Effects via 14-3-3θ in the Patient-Derived Tumor Xenograft (PDX) Model

**Figure 6:**
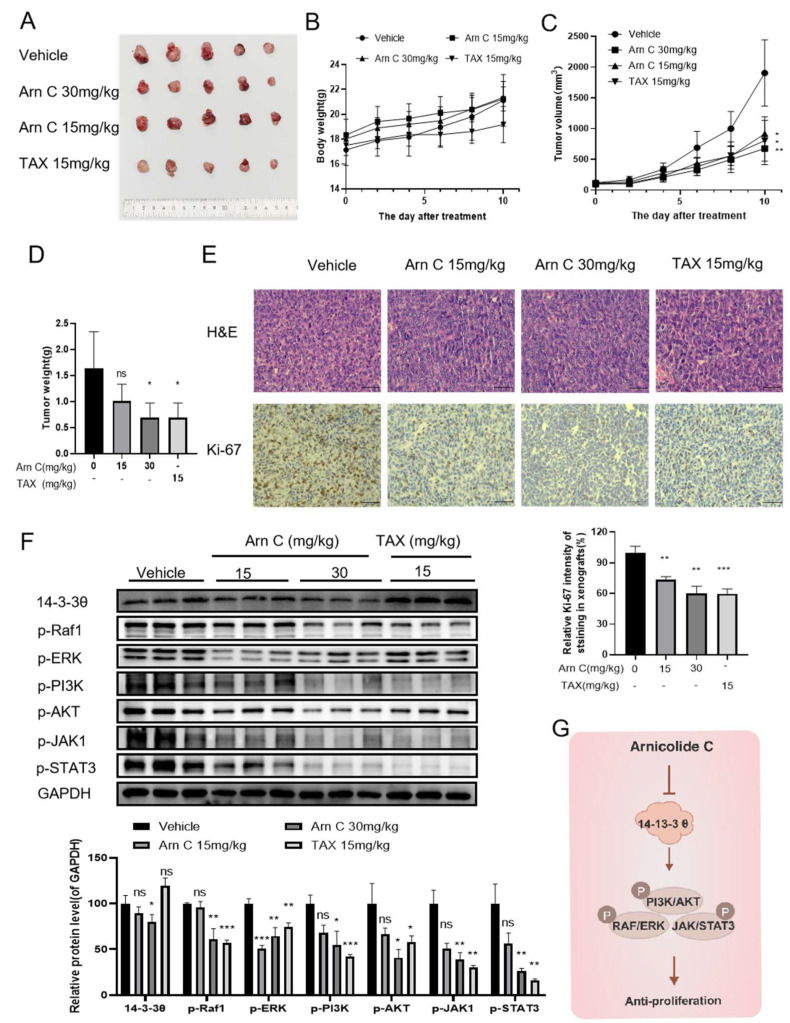
Arnicolide C inhibited patient-derived xenograft tumor growth in vivo. (**A**). Image of patient-derived xenograft tumors resected from mice. (**B**). The body weights of patient-derived xenograft tumor model mice were measured after arnicolide C (Arn C) treatment. (**C**). The volumes of patient-derived xenograft tumors. (**D**). The tumor weights of patient-derived xenograft tumors. (**E**) Representative images of staining in the vehicle group, arnicolide C (Arn C) (15 and 30 mg/kg) groups, and paclitaxel group are shown. Scale bars, 100 µm. Bar graph of the percentage of Ki-67-positive cells in tumor tissues from the vehicle group, arnicolide C (Arn C) (15 and 30 mg/kg) groups, and paclitaxel group. (**F**) Protein levels in patient-derived xenograft tumors. The protein levels of 14-3-3θ, p-Raf1, p-ERK, p-PI3K, p-AKT, p-JAK1, and p-STAT3 were measured via Western blotting. GAPDH was used as a loading control. Compared to the control group: * *p* < 0.05, ** *p* < 0.01, *** *p* < 0.001, ns (not significant). (**G**) The mechanism by which arnicolide C (Arn C) suppresses breast cancer proliferation via 14-3-3θ. A schematic representation of the relationship between arnicolide C and 14-3-3θ is shown.

To further evaluate the antitumor effect of arnicolide C in vivo and its drug development potential, we established a breast cancer PDX model (Figure 6A). Arnicolide C had no significant effect on mouse body weight in the treatment group compared with the control group (Figure 6B). Arnicolide C (30 mg/kg, i.p.) significantly inhibited tumor growth (Figure 6C,D). Moreover, we performed H&E staining and immunohistochemical staining for Ki67. The results revealed a reduced percentage of Ki-67-positive cells in breast cancer tissues after arnicolide C treatment (Figure 6E). As shown in Figure 3, arnicolide C inhibited the expression of 14-3-3θ in breast cancer cells; thus, we aimed to determine the effect of arnicolide C on 14-3-3θ and related signaling pathways in vivo. Interestingly, arnicolide C reduced 14-3-3θ protein expression in the PDX model (Figure 6F). Moreover, the levels of p-Raf 1, p-ERK, p-PI3K, p-AKT, p-JAK 1, and p-STAT3 decreased (Figure 6F). These results indicate that arnicolide C inhibited patient-derived xenograft tumor growth in vivo.

Our findings demonstrated that arnicolide C exerts antitumor effects against breast cancer by targeting 14-3-3θ both in vitro and in vivo (Figure 6G), laying the foundation for further research on this compound.

## 3. Discussion

The evaluation and mechanistic exploration of the active ingredients of traditional Chinese medicines is important for modernizing traditional Chinese medicine and developing new drugs. Arnicolide C, which is isolated from the traditional Chinese medicinal plant *C. minima*, has a tumor-suppressing effect. However, related studies in breast cancer have not been reported. Here, we report the anti-breast cancer effects of arnicolide C.

Cell cycle arrest and apoptosis are essential processes through which compounds induce tumor cell death. Compounds lead to cell cycle arrest either by directly affecting cell cycle-related proteins or indirectly affecting signaling molecules upstream of cyclins. The apoptosis pathway is an important programmed death pathway in tumor cells, and analyzing apoptosis is one of the important verification approaches to confirm the antitumor effects. The aspartate-specific cysteine protease (cysteine-containing aspartate-specific protease, Caspase) family comprises essential proteins that regulate and trigger apoptosis [49]. When cells are stimulated by apoptotic signals such as mitochondrial cytochrome c release, the activity of the apoptosis-initiating protein Caspase 9 increases, therefore initiating apoptosis [50]. In the present study, we found that arnicolide C induced cell cycle arrest and apoptosis in breast cancer cells. Its proapoptotic effect was related to the activation of Caspase9, Caspase3, and PARP-1, and the signal-triggered apoptosis may be associated with the mitochondrial apoptotic pathway. However, the specific mechanism of action of these proteins in apoptotic and signaling pathways still needs further confirmation.

The use of molecular docking to find targets bound to arnicolide C will guide further mechanistic exploration. In our study, arnicolide C was found to bind to 14-3-3θ and decrease its expression. Therefore, 14-3-3θ was recognized as a target of arnicolide C. Experimental validation of the dynamic interactions of arnicolide C with 14-3-3θ, such as molecular dynamics simulations, may be necessary. Expression at the translational level is affected by factors such as transcription and protein degradation, and further exploration is still needed to determine how arnicolide C causes a reduction in the expression level of 14-3-3θ. Since 14-3-3θ is an identified protein that promotes breast cancer proliferation and regulates related cell proliferation pathways, these findings suggest that the reduced expression level of 14-3-3θ may be a relevant molecular mechanism by which arnicolide C exerts its anti-breast cancer effect. Further studies indicated that arnicolide C may exert anti-breast cancer effects by affecting 14-3-3θ expression and thus inhibiting the downstream RAF/ERK, PI3K/AKT, and JAK/STAT pathways. However, due to its multitarget properties and complex effects, further exploration of the specific target and pathway of arnicolide C is still necessary.

Much research on the development of therapies targeting 14-3-3 proteins remains to be performed, and therapies targeting 14-3-3 have not been clinically translated. However, 14-3-3 proteins have the potential to be target proteins for antitumor therapy in the development of new drugs. Studying the biological role of 14-3-3 in the occurrence, development, invasion, and metastasis of breast cancer can aid in improving the clinical diagnosis and treatment of breast cancer in the future.

To further explore the anti-breast cancer potential of arnicolide C, an in vivo efficacy evaluation was performed using CDX and PDX models. Subcutaneous inoculation of tumor cell lines into immunodeficient mice is the most commonly used in the in vivo evaluation model in preclinical drug development and has the advantages of convenience, ease of use, and time and effort savings. Unlike in the CDX model, arnicolide C exhibited excellent tumor suppression in the PDX model, except for in the 15 mg/kg arnicolide C group. This difference may be caused by the rapid proliferation of PDXs, the more complex tumor tissue microenvironment, and the short administration period.

Our study is the first to define the biological effects of arnicolide C on inhibiting breast cancer proliferation, inducing cell cycle arrest, and promoting increased apoptosis. The anti-breast cancer efficacy of arnicolide C was further evaluated in CDX and PDX mouse models. Moreover, the molecular mechanism by which arnicolide C suppresses breast cancer was explored through molecular docking and other approaches. However, there are still several limitations of this study, and further studies are needed to reveal other biological effects of arnicolide C on breast cancer and to clarify the specific mechanism through which arnicolide C affects 14-3-3θ protein expression. We are hopeful that our results will guide the application of 14-3-3θ as a target of arnicolide C in subsequent research. Arnicolide C constitutes a new therapeutic option for breast cancer patients.

## 4. Materials and Methods

### 4.1. Chemicals and Reagents

Arnicolide C was obtained from Jiangsu Yongjian Pharmaceutical Co., Ltd. (Taizhou, China). Paclitaxel was purchased from Yangtze River Pharmaceutical Group (Taizhou, China). An apoptosis detection kit (Annexin V-PI staining kit) was purchased from Vazyme Biotech Co., Ltd. (Nanjing, China). A cell cycle detection kit was obtained from MedChemExpress (Monmouth Junction, NJ, USA).

### 4.2. Cell Culture

The Shanghai Institute of Life Science at the Chinese Academy of Sciences provided human breast cancer cells (HCC-1806, MDA-MB-468, MDA-MB-231, and SKBR3). All cell lines were authenticated by short tandem repeat (STR) profiling. MDA-MB-468 and SKBR3 cells were cultured in DMEM/F-12 medium (HyClone, Logan, UT, USA), MDA-MB-231 cells were cultured in DMEM (HyClone), and HCC-1806 cells were cultured in RPMI-1640 medium (HyClone) supplemented with 10% fetal bovine serum (FBS; Bioind, Kibbutz Beit-Haemek, Israel), 100 U/mL penicillin and 100 μg/mL streptomycin in a humidified atmosphere (Heal Force, Hong Kong, China) with 5% CO_2_ at 37 °C.

### 4.3. Cell Viability Assay

MTT passes through the cell membrane and serves as a substrate for succinate dehydrogenase in mitochondria. In viable cells, succinate dehydrogenase in mitochondria catalyzes the reduction of normally water-soluble MTT to bluish purple needle-like formazan crystals, which deposit in the cells; however, dead cells cannot reduce MTT. The resulting crystals can be dissolved in dimethyl sulfoxide (DMSO), and the color depth of the solution is proportional to the abundance of formazan. MTT assays were also conducted on breast cancer cells (HCC-1806, MDA-MB-468, MDA-MB-231, SKBR3) to determine the effects of arnicolide C. Cell suspensions were prepared, and 1800 cells per group were seeded into 96-well plates. After a 24-hour incubation period, arnicolide C was applied for 72 h, 20 μL of 0.5 mg/mL MTT solution was added, the mixture was incubated for another 4 h, and DMSO was added to dissolve the formazan. A universal microplate reader (Molecular Devices, Shanghai, China) was used to measure the absorbance at 570 nm. The inhibition rate was calculated as follows: inhibition rate (%) = (1 − absorbance of the treated well/absorbance of the control well) × 100.

### 4.4. Colony Formation Assay

Colony formation assays were conducted to determine the effect of arnicolide C on cell proliferation. A total of 2000 cells were plated and incubated for 24 h before being treated with arnicolide C for two days. The medium was then replaced with a fresh complete culture medium. After 7 days, a macroscopic count of the colonies was performed after the cells were fixed with 4% formaldehyde and stained with 0.5% crystal violet. The cell colonies were counted using ImageJ software (1.47v), and the number in each treatment group was normalized to that in the blank control group. Statistical analysis was performed, and graphs were generated using GraphPad Prism 9.0.0 software.

### 4.5. Apoptosis Detection and Cell Cycle Analysis

EDTA-free trypsin was used to collect cells, and ice-cold PBS was used to wash them. Subsequently, the cells were suspended in 500 mL of binding buffer and stained with PI and FITC-conjugated Annexin V for 15 min. Apoptotic cells were identified with a FACSCalibur flow cytometer (BD Biosciences, San Jose, CA, USA).

Propidium iodide (PI) binds to double-stranded DNA to produce fluorescence, and the fluorescence intensity is proportional to the content of the double-stranded DNA. Since the DNA content of cells varies during cell division, PI can distinguish cells in different cell cycle phases. For cell cycle analysis, PI staining was performed after cells were collected, fixed in 75% ethanol overnight, and washed once with ice-cold PBS. The cells were then stained with PI at 37 °C for 30 min. Cell cycle analysis was performed using a FACSCalibur flow cytometer (BD Biosciences).

### 4.6. Western Blotting

We performed Western blotting according to the protocol described in a previous study [51]. The following antibodies were purchased from Cell Signaling Technology (Danvers, MA, USA) for Western blotting: anti-cleaved Caspase3 (Cat#9661), anti-cleaved Caspase9 (Cat#9509), anti-cleaved PARP-1 (Cat#9541), anti-JAK1 (Cat# 3344), anti-p-JAK1 (Cat#74129), anti-STAT3 (Cat#9139), anti-p-STAT3 (Cat#9145), anti-p-PI3K (Cat#4228), anti-p-RAF1 (Cat#9427), anti-p-ERK (Cat#4370), anti-AKT (Cat#4691), and anti-p-AKT (Cat#4060). The anti-14-3-3-θ (Cat# ab183075) antibody was purchased from Abcam (Cambridge, UK). The anti-GAPDH (Cat#AC002) antibody was purchased from ABclonal Technology (Wuhan, China). HRP-conjugated anti-rabbit IgG (Cat#AS014) and HRP-conjugated anti-mouse IgG (Cat#AS003) (ABclonal Technology, Wuhan, China) were used as secondary antibodies, and enhanced chemiluminescence reagent (Vazyme, Nanjing, China) was used for detection of signals after exposure of the membrane in an image analyzer (Bio-Tanon, Shanghai, China).

### 4.7. Molecular Docking

The 14-3-3θ protein structure used for molecular docking was obtained from the PDB database. Docking validation was performed using MOE software (2019.0102).

### 4.8. Nude Mouse Xenograft Study

Female BALB/c athymic nude mice (6 weeks old) with body weights ranging from 16 to 20 g were purchased from Huafukang Bio. A total of 5 × 10^6^ MDA-MB-468 cells were injected subcutaneously into the axilla. Tumors were allowed to grow in subcutaneous tissues to a volume of 100 mm^3^. Mice were divided randomly into 4 groups of 6 individuals each. Arnicolide C was administered intraperitoneally at a concentration of 15 mg/kg or 30 mg/kg daily. Mice in the negative control group were administered an equal amount of normal saline. Mice in the positive control group were administered 15 mg/kg paclitaxel by tail vein injection twice weekly. When the mean tumor volume in the control group reached approximately 1500 mm^3^, the mice were euthanized, and the tumor tissues were then resected and assessed. The tumor volume (TV) was calculated by the following equation: TV (mm^3^) = A × B^2^/2, where A is the longest diameter of the tumor and B is the shortest diameter. To calculate the relative tumor volume (RTV), we used the following equation: RTV = V_t_/V_0_, where V_t_ is the TV on day t and V_0_ is the TV on Day 0. The Animal Care and Control Committee of Jiangsu Hanjiang Biotechnology Co., Ltd (Taizhou, China). guided the animal care and surgical procedures.

### 4.9. Patient-Derived Tumor Xenograft Study

Tumor tissue with necrosis and connective tissue in human breast cancer tumor tissue was excluded, and high-quality tumor tissue was retained. Human breast cancer tumor tissue was sheared to generate pieces of approximately 1 mm^3^ in volume, and these pieces were implanted into the renal capsule of BALB/c nude mice using a trocar. After the tumors grew, part of the tumor tissue was cut into 1 mm^3^ pieces, which were inoculated subcutaneously into the right forelimb axillary region of BALB/c nude mice using a trocar. The remaining tumor tissue was cut into small pieces and stored frozen in liquid nitrogen. After the third generation of tumors grew, the tumor tissue was inoculated subcutaneously into the axilla of the right forelimb of the mice needed for the experiment. The drug treatment groups were established when the tumor volume reached approximately 100 mm^3^. The mice were randomly divided into four groups. Arnicolide C or paclitaxel was administered. The follow-up treatment was the same as described above (Section 4.8). A portion of the tumor tissue was used for protein expression analysis, and other portions were fixed in paraformaldehyde for H&E staining and IHC staining. The animal care and surgical procedures followed the guidelines of the Animal Care and Control Committee of Jiangsu Hanjiang Biotechnology Co., Ltd. Patient ethics and informed consent were reviewed and approved by the Clinical Research Ethics Committee of Jiangsu Taizhou People’s Hospital.

### 4.10. Statistical Analyses

All the data in the study are expressed as the mean ± S.D. values and were analyzed using one-way ANOVA (* *p* < 0.05; ** *p* < 0.01; *** *p* < 0.001; ns, no significant difference).

## 5. Conclusions

In summary, we evaluated the inhibitory effects of arnicolide C on breast cancer. Arnicolide C inhibited the growth of HCC-1806 and MDA-MB-468 cells both in vitro and in vivo, mainly by inducing apoptosis and G1 arrest. Furthermore, 14-3-3θ was confirmed to be a target of arnicolide C. Furthermore, these findings demonstrated that arnicolide C inhibited the expression of 14-3-3θ, which is involved in the regulation of proliferation-related pathways, including the RAF/ERK, PI3K/AKT, and JAK/STAT pathways.

## Data Availability

All relevant data are provided within the paper and its supplementary materials. The data supporting the findings of this study are available from the corresponding author upon reasonable request.

## Supplementary Materials

The following supporting information can be downloaded at: https://www.mdpi.com/article/10.3390/ph17020224/s1.
