# Supplementary figures and images for: Arnicolide C Suppresses Tumor Progression by Targeting 14-3-3θ in Breast Cancer
Source: Pharmaceuticals (Basel). 2024 Feb 8;17(2):224. doi: 10.3390/ph17020224 (PMC10892132; doi:10.3390/ph17020224)

Fig 2E-F

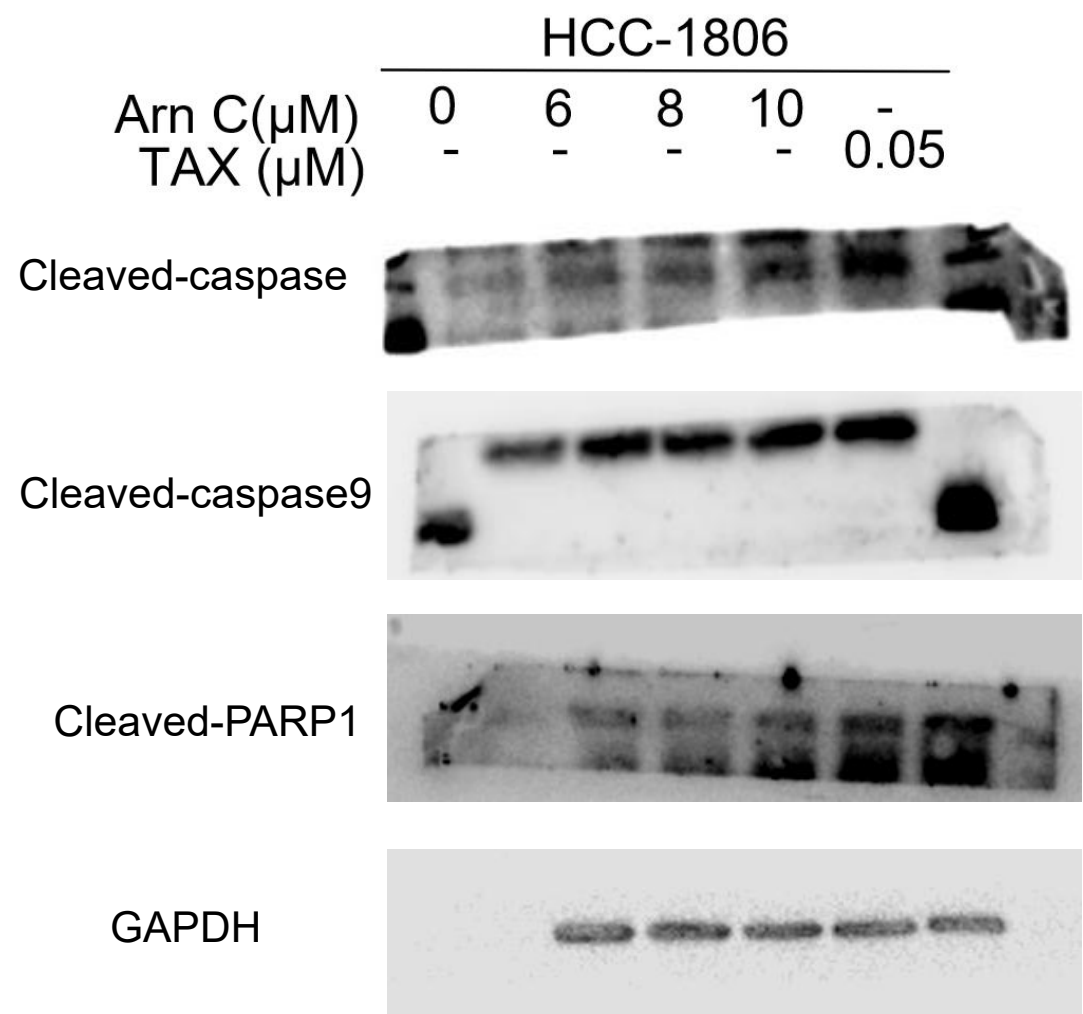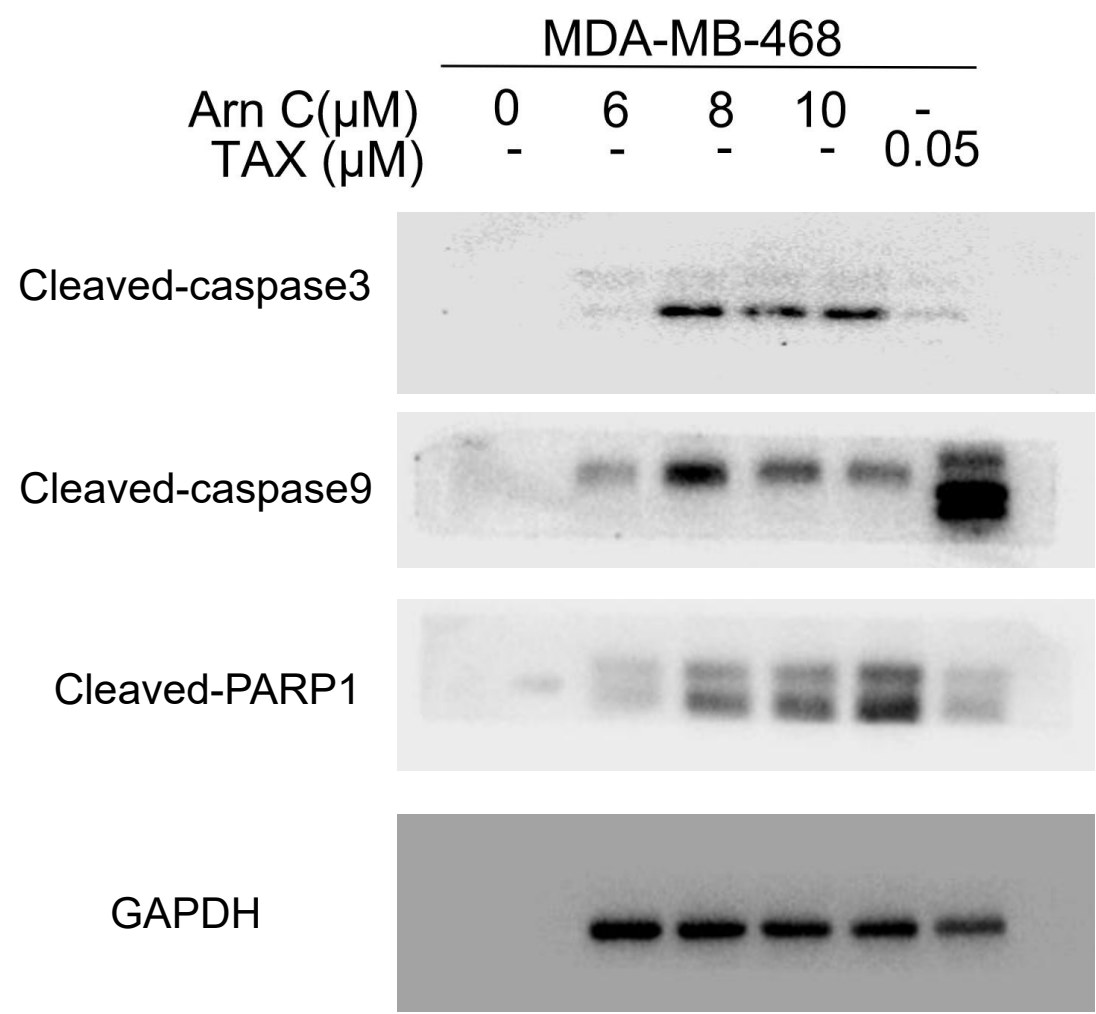

**Fig 3C**

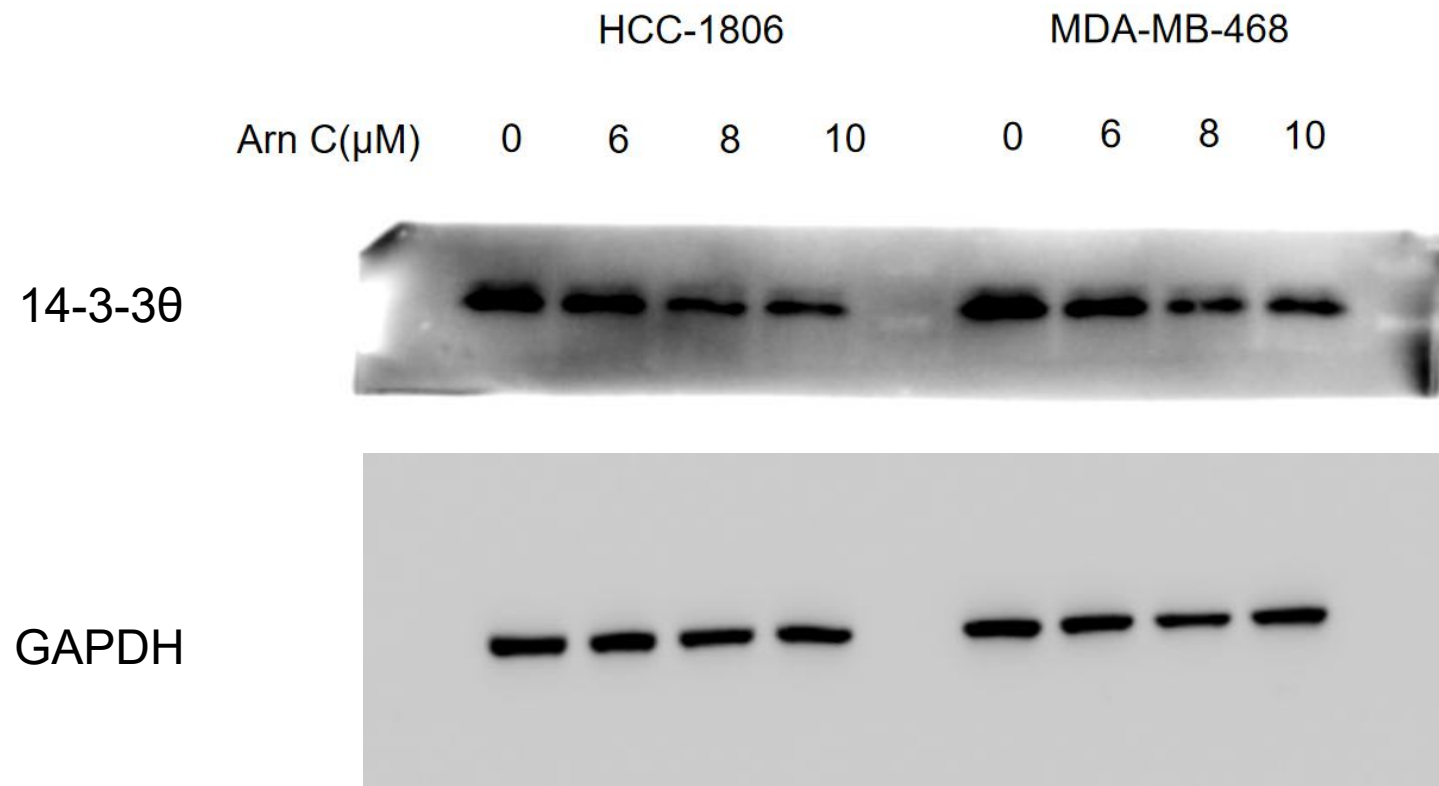

Fig 4A

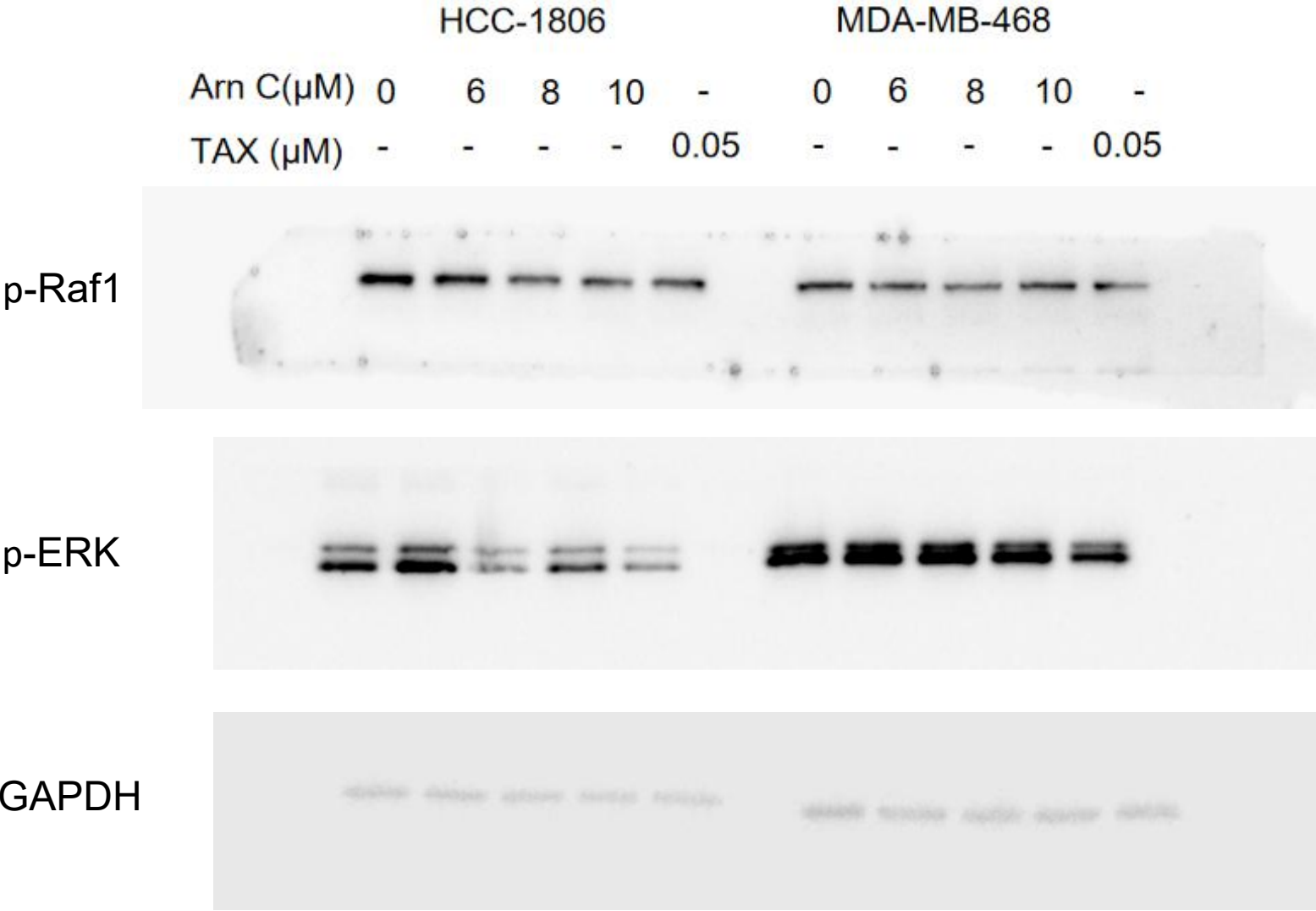

Fig 4B

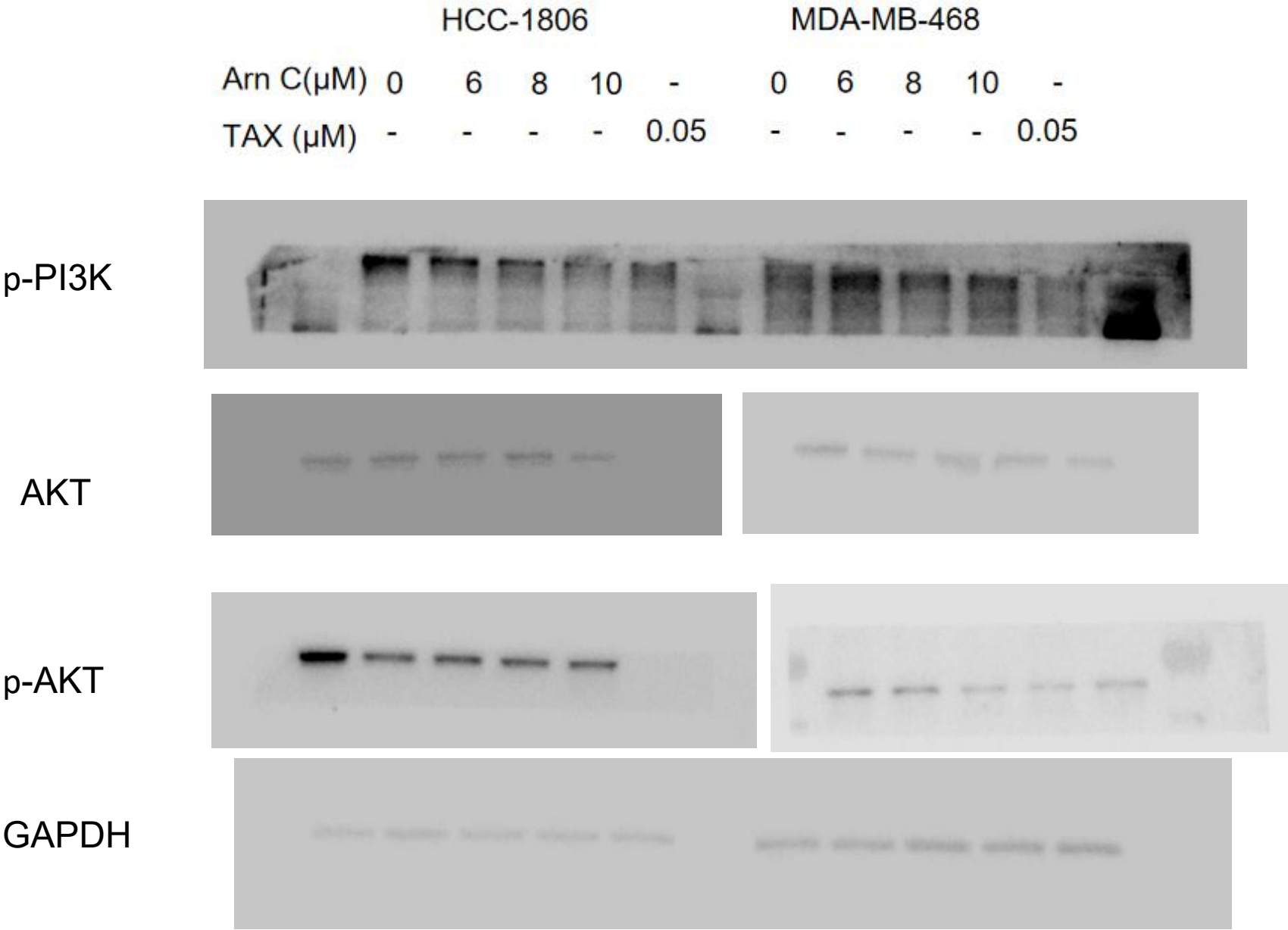

Fig 4C

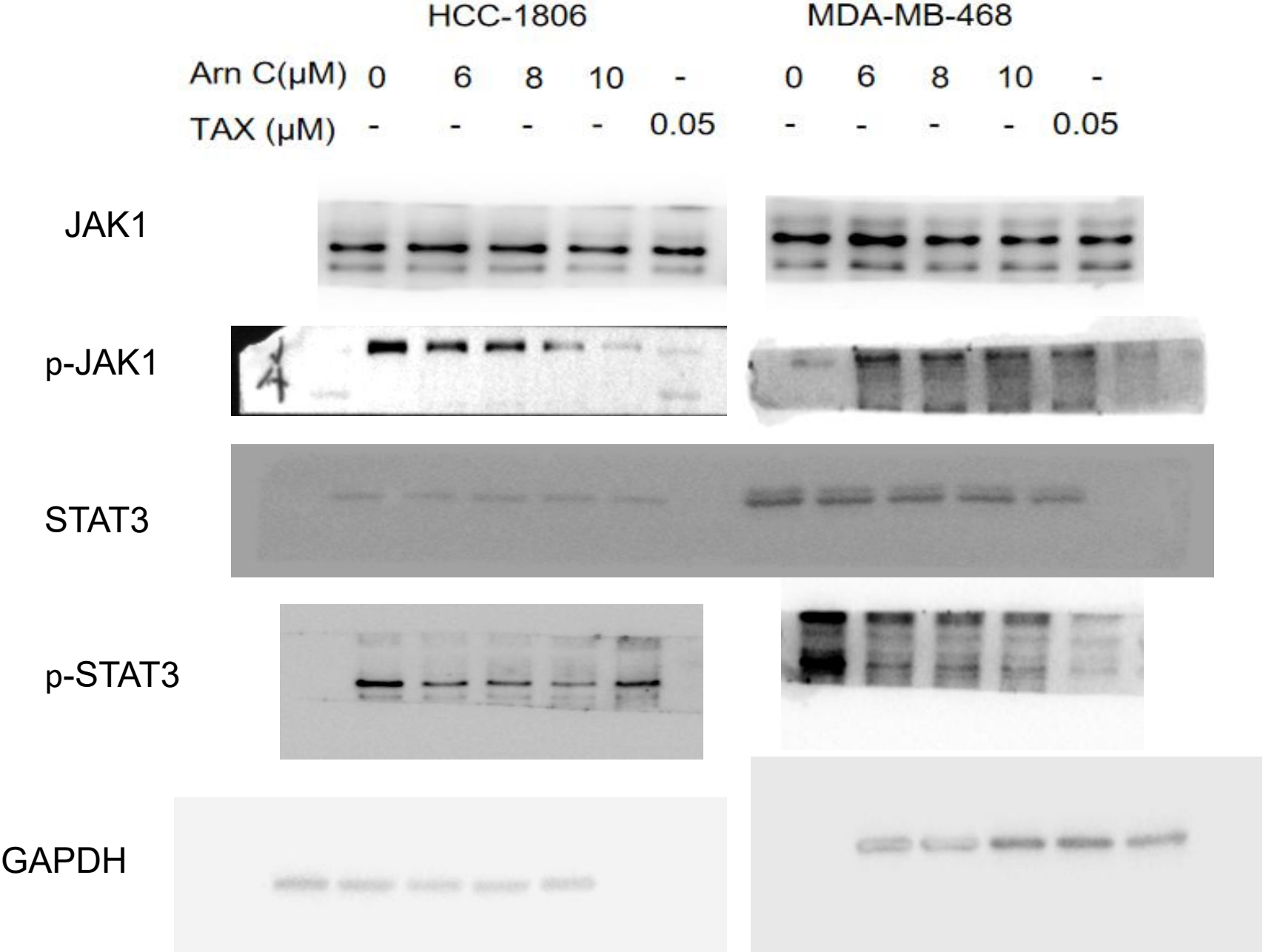

Fig 6F

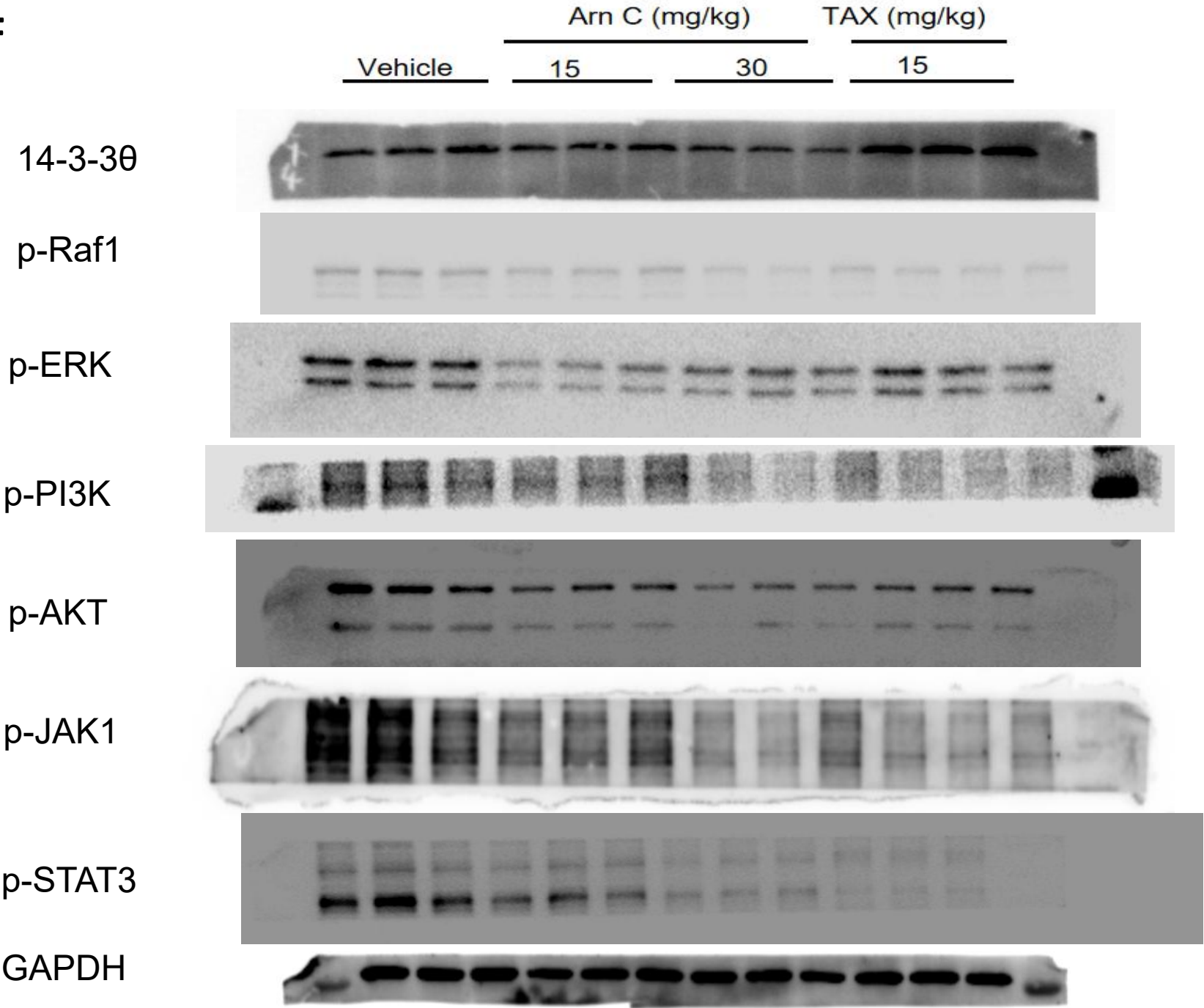

Supplement: Supplementary file 1 [file pharmaceuticals-17-00224-s001.zip › pharmaceuticals-2815816-supplementary.pdf]
